# Progression from an internal pilot of a main trial including adaptation from a two to three group study - straightforward to do?

**DOI:** 10.1186/1745-6215-16-S2-O17

**Published:** 2015-11-16

**Authors:** Danielle Edwards, Graziella Mazza, Rachael Heys, Caroline Wilson, Jane Blazeby, Chris Rogers

**Affiliations:** 1 University of Bristol, Bristol, UK

## 

An internal pilot phase of a trial is often undertaken to establish recruitment and retention. The decision criteria at the juncture of trial progression also provide an opportunity for adaptation if pre-specified in the design. Adaptation can increase trial efficiency and relevance. We present methods to justify and implement adaptation from a two to 3-group trial at progression from an internal pilot to main trial.

A multi-centre RCT with an internal pilot to establish recruitment also included plans to consider adaption from a two to three-group trial at the time of progression. Plans to expand the trial were based on analysis of new evidence and on-going RCTs and scrutiny of current NHS and private practice to understand rates of the new and existing interventions.

Recruitment in the pilot phase was successful. Systematic review evidence and analysis of trials did not reveal competing studies or new evidence. Practice data showed similar incidence of all three interventions supporting the need for an adaption. The revised and expanded 3-group trial with updated hypotheses was funded meaning that modification of the protocol, patient-related documents and study database was required. The randomisation system had to move from two to 3-groups and provide three balanced groups at the end of the trial. Study website and publicity materials were adapted to account for the three groups.

Implementing the adaptation, while recruitment to the trial continued was challenging and not to be underestimated. This approach allows an existing trial to maintain research of relevance to practice.

